# Information About Canadian Patient Groups’ Conflicts of Interest and Industry Funding—Incomplete, Inconsistent, and Unreliable: A Cross-Sectional Study

**DOI:** 10.1177/27551938251325801

**Published:** 2025-03-17

**Authors:** Joel Lexchin

**Affiliations:** 1 School of Health Policy and Management, 7991 York University, Toronto, Canada; 2 Faculty of Medicine, 7938 University of Toronto, Toronto, Canada

## Abstract

Patient groups play an important role in health care. At the same time, the majority of Canadian groups receive payments from pharmaceutical companies, which calls into question whether they speak for the best interests of their membership or the companies that fund them. Canada lacks any mandatory reporting by either patient groups or pharmaceutical companies regarding payments between groups and companies. There are three potential sources of information on the topic of payments: (*a*) declarations made by groups when they file submissions to the Canadian Agency for Drugs and Technologies in Health, an organization created and funded by Canada's federal, provincial and territorial governments, about whether the agency should recommend public funding for the new drug; (*b*) patient groups’ websites; and (*c*) voluntary disclosures by pharmaceutical companies on their websites. This study investigates the data available in all three sources and finds that they are incomplete and inconsistent, making any conclusions about patient groups’ conflicts of interest and funding unreliable. Although increased transparency is no guarantee of independence, it is an important and necessary first step. However, relying on voluntary disclosure is not sufficient. Legislation, such as the bill passed in the province of Ontario but never implemented, mandating disclosure by companies of payments that they have made is necessary.

Patient groups should play an increasingly important role in health care both in Canada and internationally through advocating for the interests of their members, promoting the visibility of their conditions, pushing for more rapid and accurate diagnoses, and lobbying for the introduction and funding of new treatments and drugs that may help relieve their members’ symptoms and extend their lives. However, groups in Canada often face significant structural barriers to meaningful representation and engagement, one of the most prominent of these being the lack of funding. Since the Canadian federal government rolled back funding of patient groups in the mid 1990s, groups have had to seek new sources of revenue, and many of them now rely on pharmaceutical companies. The availability of funding has increased interactions between the two^1^ and the acceptability of industry funding by patient groups. Seventy-two percent of the 100 Canadian patient groups who responded to a 2023–2024 survey viewed pharmaceutical companies as having an excellent or good reputation, although there were criticisms about their pricing policies.^2^

In Europe, research into the funding of patient organizations is enabled by the Code of Practice of the European Federation of Pharmaceutical Industries and Associations (EFPIA), which sets out minimum requirements that companies have to follow in declaring funding. In particular, each member company must annually disclose on its website “a list of POs [Patient Organizations] to which it provides financial support and/or significant indirect/non-financial support or with whom it has engaged to provide contracted services for that Member Company”.^3^ While research has found that these requirements fail to adequately reveal the extent of funding of patient groups in the United Kingdom^4^ and the Nordic countries,^5^ they are still superior to the position of Innovative Medicines Canada (IMC), the Canadian equivalent of EFPIA, in its Code of Ethical Practices, which merely encourages Member Companies “to appropriately acknowledge support they provide [to patient organizations] and to ask recipients to do so”.^6^ Thus, from the perspective of the Canadian industry, disclosure rules are almost nonexistent, and virtually nothing can be gleaned about disclosure practices and disclosure data.

From the perspective of patient groups, information on their funding by pharmaceutical companies is equally difficult to obtain. Seventy-nine of 116 patient groups disclosed receiving a total of 373 donations from companies belonging to the industry lobby group IMC, but almost one third did not report receiving donations from companies that claimed that they donated to them.^7^ Most patient groups are registered charities and file annual financial reports to the Canada Revenue Agency, but those publicly available reports do not contain detailed information about their source of funding. See, as an example, the financial report for Canadian Cancer Survivor Network (https://apps.cra-arc.gc.ca/ebci/hacc/srch/pub/dsplyRprtngPrd?q.srchNm = canadian + cancer + survivor + network&q.stts = 0007&selectedCharityBn = 834540882RR0001&dsrdPg = 1). There is also no legislation or other rules mandating that patient groups post the names of commercial companies that provide funding to them or the amount of money that they receive on their websites or report this information in any other way. Several groups voluntarily disclose some of this information.

The Canadian Agency for Drugs and Technologies in Health (CADTH, now Canada's Drug Agency), an organization created and funded by Canada's federal, provincial and territorial governments, undertakes health technology assessments for all federal, provincial and territorial drug plans, except for Quebec, and recommends whether drug indications (hereafter drugs) should be funded. Previous work has found that virtually all patient groups that made submissions to CADTH about public reimbursement had some funding from drug companies. Further, only one percent of submissions opposed funding.^8^ (Although CADTH has recently changed its name, the abbreviation CADTH will be used in this article.) CADTH encourages patient groups to make representations about whether the organization should recommend funding of drugs to public plans. However, making submissions is conditional on the groups disclosing any real, potential, or perceived conflicts of interest (COI) within the previous two years that they have with companies or organizations that may have a direct or indirect interest in the drug under review.^9^

Internationally, the findings about the lack of transparency by patient groups about their funding from industry are similar to those in Canada. A study of groups in the United Kingdom concluded that recipients underreported payments to the extent that the number and value of payments reported by donors were 259.8 percent and 163.7 percent, respectively, greater than those reported by recipients.^10^ Elsewhere, researchers found that disclosure of funds was scarce on Italian patient and consumer groups’ websites.^11^ A systematic review of the literature on the financial relationships between patient groups and the health sector industry reported that among organizations for which there was evidence of industry funding, only a median proportion of 29 percent acknowledged receiving that funding on their websites.^12^

Many patient groups might have difficulty functioning without industry funding, but a lack of transparency about the source of their revenue and what percent of their budget comes from that source can compromise patient organizations’ independence and credibility.^13–15^ This study sought to determine if it was possible to obtain accurate information on patient groups’ COI with drug companies, including the amount of revenue they receive and how much of their total budget that revenue source represents.

## Methods

This cross-sectional analysis uses information about COI and payments from three sources: declarations made by patient groups to CADTH, patient groups’ websites, and companies’ websites. It compares the level of disclosure about patient groups’ relationships with and revenue from pharmaceutical and medical device companies (hereafter pharmaceutical companies) coming from each of the three sources of information.

### Submissions to CADTH

In order to try to ensure that the information on patient groups’ websites about company funding as of June 29, 2024 was consistent with patient groups’ disclosures to CADTH, only reports^16^ of drugs considered by CADTH that met the following criteria were analyzed: drug industry submissions were accepted by CADTH after January 1, 2022 and recommendations were finalized prior to when data gathering for this study was finished (June 29, 2024). According to CADTH, patient groups are supposed to disclose COI in the two years prior to a submission, which for the purpose of this study was interpreted as two years prior to the date when patient group submissions for a particular drug closed. (The CADTH Reimbursement Review Patient Input Template that patient groups use to report COI can be downloaded using the hyperlink on page 65 of reference 9.) The following information from the CADTH Clinical, Pharmacoeconomic, and Stakeholder Input Combined Report was extracted and entered into an Excel spreadsheet: generic and brand name, name of submission sponsor (pharmaceutical company), indication, the date that CADTH accepted the submission, the date when patient group submissions closed and the final CADTH recommendation (reimburse, reimburse with clinical criteria or conditions, do not reimburse).

### Patient Group Information

From the same Combined Report mentioned above, the following information was abstracted: name of the patient group commenting on the company submission, name of the companies the patient group declared a COI with, amount of money received from each pharmaceutical company (grouped by CADTH into the following categories: $0–$5,000, $5,001–$10,000, $10,001–$50,000, $50,000+).

Patient groups were defined as those whose primary mission was to combat a particular disease or disability or to work toward improving the health and well-being of a particular patient population, a definition broadly similar to the one used by Colombo and colleagues in their study of industry funding of Italian groups.^11^ In line with this definition of a patient group, donations to other organizations, for example, foundations whose primary focus was on fund raising for a particular disease, were not included. If there were doubts about whether an organization was a patient group based on its description in the CADTH report, the patient group's website was identified and the description of the organization on the website was read in detail to confirm that it fit the a priori definition of a patient group. If two or more patient groups collaborated on a single submission they were treated separately if each group made its own COI declaration and as one group if there was one COI declaration for both groups.

On occasion, patient groups declared receiving payments from the same company in two different years in the same submission to CADTH. In these cases, the amounts were added and only a single payment was recorded. If separate payments were made from a parent and subsidiary company, the payments were recorded as coming from just a single company. Similarly, if separate payments were made by the Canadian and international branches of the same company, they were recorded as coming from a single company.

If it was unclear whether a company making a payment fit the definition of a pharmaceutical company, the website of the company was researched online and the description of the company on the website was read in detail. If the company was found to produce, distribute or market pharmaceutical products (including small molecule drugs and/or biologics), it was considered a pharmaceutical company. Companies only producing items such as medical software, diagnostic kits, natural health products or food supplements were excluded.

### Patient Groups’ Website Information

The website of each patient group making a submission to CADTH was initially manually searched for a list of company sponsors and, if present, the amount of money the company provided to the patient group. Both the company name and the amount of money were recorded on the spreadsheet. Only information from the websites of Canadian patient groups was used on the grounds that patient groups headquartered in other countries may have different requirements for reporting payments. If no donor names were found on the manual search, then a Google search of the website was done by going to Google.com and entering < site:> followed by the URL of the main domain, then < a space > and finally the search term. The search term consisted of the name(s) of the company that the group reported receiving money from when it made its submission to CADTH. If the group did not report receiving money from a company, then the website was manually searched two additional times. All information was recorded on the spreadsheet. Websites were also searched for audited financial reports that reported the total annual revenue of the group. Reports from the most recent year (up to and including 2023) were used and the amount of revenue and the URL for the location of the report were recorded on the spreadsheet.

Patient groups were not contacted about the names of pharmaceutical company sponsors and the amount of money that they receive from them on the grounds that this information should be publicly available and easily located by anyone interested in the information.

### Companies’ Website Information

A list of the 20 top pharmaceutical companies by worldwide revenue in 2023 was downloaded^17^ and a Google search was done for each of the companies to determine if they disclosed funding to Canadian patient groups. If such a list was found, it was searched for the name of patient groups that had made submissions to CADTH. Names of the groups were downloaded along with the amount of money that they received. If the payments were recorded in a foreign currency, it was converted into Canadian dollars using the Bank of Canada annual exchange rate for the appropriate year.^18^

### Data Analysis

Medians and interquartile ranges (IQR) were computed as necessary using the function on Excel.

The Anatomic-Therapeutic-Chemical (ATC) 2nd level designation was recorded for each drug evaluated by CADTH in order to differentiate antineoplastic drugs from drugs indicated for other conditions^19^ and counts were made of the number of drugs in each of the ATC 2nd levels.

Counts were also made of the following information: number of drugs considered by CADTH, number of companies making a submission, number of different submissions made by each company (i.e., one company could have made multiple submissions for different drugs), CADTH recommendations, number of patient groups making submissions for each drug and number of unique submissions made by each patient group (i.e., one patient group could have made multiple submissions for different drugs), and total number of COIs with individual companies declared by each group in all their submissions.

An estimate was made of the amount of money received by each patient group from companies using three different methods. First, all the submissions to CADTH made by an individual patient group were combined along with the payment amounts from each company. Next, to avoid double counting, any duplicate payments from the same company in the same year for the same amount of money were disregarded. Finally, payments recorded in each of the CADTH categories were summed using the following formula: $2,500 (payment between $0–$5,000), $7,500 (payment between $5,001–$10,000), $30,000 (payment between $10,001–$50,000), $50,000 (payment $50,000+).

Second, counts were made of the number of companies that individual patient groups reported receiving funding from on their websites. If there was information about the value of the payments, then this was used to estimate the revenue from company sources. If the amount of money was reported in categories, then the same formula used for payments reported to CADTH was also used, that is, taking the middle value from each category and using the starting amount of the highest category.

The third method of estimating payments from companies was to sum payments to individual patient groups that companies listed on their websites.

The number and name of companies listed by the same patient group in different CADTH submissions were examined for similarities and differences. Patient group information from their CADTH declarations and from their websites was compared regarding the value of payments and the name of companies that provided the payments. The value of payments reported by companies on their websites was also compared to the value of payments calculated from patient groups’ CADTH declarations and from patient groups’ websites.

The percent of patient groups’ revenue coming from company sources was estimated using the total revenue reported in audited financial reports and industry payments reported in each of the three sources: the sum from CADTH declarations, the amount listed on patient groups’ websites, and the amount reported by companies. These three different estimates were compared.

### Data Gathering, Patient Involvement, Ethics

All data were gathered by a single individual between June 24–29, 2024. No patients or patient groups were involved in this study. Ethical approval was not sought as all data were publicly available. This study follows the STROBE reporting guidelines.

## Results

### Pharmaceutical Companies’ Submissions to CADTH

Between January 19, 2022 and September 13, 2023 CADTH published reports on its website for 102 drugs that had input from patient groups. Submissions to CADTH came from 36 individual companies making between one and nine submissions each, median = 2 (IQR 1, 4). In three cases the reimbursement review was requested by public drug plans and these reviews were not analyzed (see Table 1). Each company submission had comments from one to seven patient groups, median = 2 (IQR 1, 2). CADTH recommended no reimbursement in 12 cases and reimbursement with conditions or clinical criteria in the other 90 submissions. (There were no recommendations to reimburse without conditions or clinical criteria.) Forty-nine drugs were antineoplastic agents, 17 were immunosuppressants, and the remaining 36 were in 22 other ATC categories. (One drug had not been assigned an ATC group.) All three ATC groups of drugs had a median of one indication per drug (IQR 1, 1). Supplementary Table 1 presents all the data gathered from the CADTH reports, the World Health Organization website, and patient groups’ websites.

**Table 1.** Pharmaceutical Companies Making Drug Indication Submissions to CADTH.

Name of company	Number of drug indication submissions
AstraZeneca	9
AbbVie (including Allergen)	8
Hoffmann La-Roche	8
Merck	7
Bristol Myers Squibb (including Celgene)	6
Janssen	6
Novartis Pharmaceuticals (including Advanced Accelerator Applications)	5
Alexion Pharma	4
Gilead Sciences	4
Sanofi-Aventis	4
Bayer	3
Ipsen Biopharmaceuticals	3
Non-sponsored	3
Otsuka (including Taiho)	3
Alnylam Netherlands	2
Eli Lilly	2
GlaxoSmithKline	2
Jazz Pharmaceuticals	2
Medison	2
Novo Nordisk	2
Arcutis Biotherapeutics	1
Argenx	1
BeiGene	1
BioCryst Pharmaceuticals	1
Boehringer Ingelheim	1
Chiesi	1
Eisai	1
FORUS Therapeutics	1
Lundbeck	1
Medexus Pharmaceuticals	1
Pfizer	1
Rhythm Pharmaceuticals	1
Servier	1
SOBI	1
Takeda	1
Ultragenyx Pharmaceutical	1
Vertex	1
Total (Median, IQR)	102 (2, IQR 1, 4)

### Patient Groups Submissions to CADTH

Seventy-four patient groups made 183 submissions to CADTH (median = 2, IQR 1, 3). Three of the submissions were about drugs that public drug plans requested CADTH to evaluate, leaving 180 patient group submissions for analysis. Sixty-two groups declared a total of 327 conflicts (median = 2, IQR 1, 3) in those 180 submissions. The maximum number of COI from a single group was 21 in two submissions. (Multiple groups named the same companies.) Nine groups explicitly declared that they did not have any COI and three groups did not mention COI in their submissions (see Table 2). Patient groups disclosed COI with companies either in their CADTH declarations or on their websites in 156 (86.7%) of the 180 submissions they made. Based on CADTH's requirement to declare COI within the previous two years, COI occurred between January 28, 2020 and September 1, 2023.

**Table 2.** Patient Groups: Number of CADTH Submissions, Number of Conflicts-of-Interest and Estimated Revenue from Pharmaceutical Companies.

Patient group name	Number of separate submissions to CADTH	Number of COI with pharmaceutical companies declared in all submissions to CADTH	Patient group revenue from pharmaceutical companies (estimated from CADTH declarations) (CAN $)
Canadian Breast Cancer Network	8	9	440,000
Leukemia & Lymphoma Society of Canada	8	4	155,000
Canadian Cancer Survivor Network	7	6	387,500
Gastrointestinal Society	7	5	350,000
Lung Cancer Canada	7	4	162,500
Lymphoma Canada	7	8	370,000
Rethink Breast Cancer	7	5	300,000
Canadian Organization for Rare Disorders	5	5	100,000
Crohn's and Colitis Canada	5	3	150,000
Diabetes Canada	5	15	2,110,000
Kidney Foundation of Canada	5	12	427,500
Arthritis Consumer Experts	4	0 (no COI declared)	N/A
Canadian Council of the Blind	4	2	100,000
Canadian Skin Patient Alliance	4	11	422,500
Fighting Blindness Canada	4	4	200,000
HeartLife Foundation	4	6	257,500
Lung Health Foundation / Ontario Lung Association	4	4	132,500
Arthritis Society Canada	3	17	365,000
Asthma Canada	3	8	350,000
Canadian Arthritis Patient Alliance	3	4	42,500
Canadian Association of Psoriasis Patients	3	12	265,000
Canadian Liver Foundation	3	5	230,000
Canadian Psoriasis Network	3	13	422,500
Colorectal Cancer Resource & Action Network	3	2	60,000
CreakyJoints Canada	3	1	30,000
Migraine Canada	3	6	260,000
Save Your Skin Foundation	3	2	100,000
Aplastic Anemia & Myelodysplasia Association of Canada	2	6	112,500
CLL Canada	2	3	35,000
Colorectal Cancer Canada	2	17	930,000
Kidney Cancer Canada	2	6	297,500
Lupus Canada	2	2	60,000
Melanoma Canada	2	2	100,000
Migraine Quebec	2	8	230,000
Mood Disorders Society of Canada	2	6	270,000
Muscular Dystrophy Canada	2	2	100,000
Myeloma Canada	2	21	787,500
VTE-COS Patient Partners	2	5	65,000
aHUS Canada	1	0 (no COI declared)	N/A
Answering Thrombotic Thrombocytopenic Purpura Foundation	1	0 (no COI declared)	N/A
Bardet Biedl Syndrome Foundation	1	1	50,000
Bladder Cancer Canada	1	0 (no COI declared)	N/A
Breast Cancer Canada	1	2	100,000
British Columbia Lung Association and Lung Groups	1	5	103,500
Canadian Association of PNH Patients	1	4	120,000
Canadian Chronic Myelogenous Leukemia Network	1	1	7,500
Canadian FOP Network	1	0 (no COI declared)	N/A
Canadian Heart Patient Alliance	1	0 (no COI declared)	N/A
Canadian Hemophilia Society	1	8	362,500
Canadian Neuroendocrine Tumour Society	1	4	72,500
Canadian Spondylitis Association	1	7	290,000
Canadian VHL Alliance	1	0 (no COI declared)	N/A
Chronic Myelogenous Leukemia Society of Canada	1	0 (no COI declared)	N/A
Cystic Fibrosis Canada	1	5	120,000
Eczéma Québec	1	1	30,000
Eczema Society of Canada	1	1	50,000
HAE Canada	1	6	240,000
HPV Global Action	1	6	147,500
Lupus Ontario	1	1	30,000
Menopause Chicks	1	1	2,500
MS Canada	1	12	412,500
Obesity Canada and the Canadian Liver Foundation	1	1	50,000
Obesity Matters	1	1	2,500
Oxalosis & Hyperoxaluria Foundation	1	1	50,000
Parkinson Association of Alberta	1	1	7,500
Parkinson Canada	1	1	50,000
Sickle Cell Awareness Group of Ontario	1	0 (no mention of COI in declaration)	N/A
Thalassemia Foundation of Canada	1	0 (no mention of COI in declaration)	N/A
Thyroid Cancer Canada	1	0 (no COI declared)	N/A
TTR Amyloidosis Canada	1	0 (no mention of COI in declaration)	N/A
Tumour Foundation of BC	1	1	2,500
Vasculitis Foundation Canada	1	1	30,000
Waldenstrom's Macroglobulinemia Foundation of Canada	1	1	50,000
Women's Health Coalition of Alberta Society	1	3	22,500
Total (median, IQR)	183 (2, IQR 1, 3)	327 (2, IQR 1, 3)	13,581,000 (median 120000, IQR 50000, 298125)

The estimated total revenue from pharmaceutical companies for the 62 patient groups that made COI declarations in their CADTH submissions was $13,581,000; the median was $120,000 (IQR 50,000, 298,125) with a range of $2,500 to $2.11 million per group. One group (Diabetes Canada) gave more precise ranges of the value of payments it received in its declaration with a top amount of $400,000 + and ranges of $100,000–$399,999, $50,000–$99,999, $10,000–$49,999 and under $10,000.

Supplementary Table 2 compares COI declarations for patient groups that made more than one submission to CADTH. The list of companies was similar in separate declaration from many groups with minor variations but in some cases, there were significant differences. For example, the Leukemia & Lymphoma Society of Canada made eight separate submissions; in two it declared no conflicts and in each of the other six, it named a different company. The Kidney Foundation of Canada made five separate submissions and in four declared nine or ten COI, but in the fifth did not mention the subject of COI. Myeloma Canada declared 22 COI in one submission but only 14 in a second.

### Patient Groups’ Websites

Forty-eight (64.9%) of the 74 patient groups listed pharmaceutical company sponsors on their websites, with a range of 1 to 31 companies (median = 8, IQR 5, 12) for a total of 457 sponsorships (see Table 3). Multiple groups named the same companies. Nine groups categorized their sponsors using terms such as “main partner,” “global sponsor,” and “arthritis champions,” but no monetary value was attached to these categories. Three groups provided enough information to estimate the total value of payments from pharmaceutical companies: $710,000 (Diabetes Canada), $543,950 (Eczema Society of Canada) and $1,290,000 (Lung Health Foundation/Ontario Lung Association (see Table 3). No group explicitly stated the exact value of payments that only came from pharmaceutical companies. Six groups provided vague descriptions of how the payments were used, for example, producing booklets and funding awareness programs, but no group stated the use of individual payments from companies. Two groups stated that the grants they received were unrestricted (see Table 4).

**Table 3.** Number of Pharmaceutical Company Sponsors Listed on Patient Groups’ Websites and Monetary Value of Payments from Companies.

Patient group	Number of pharmaceutical company sponsors listed on groups’ website	Monetary value of payments based on information on patient groups’ websites (CAN $)
Canadian Organization for Rare Disorders	31	N/A
Colorectal Cancer Canada	21	N/A
Canadian Liver Foundation	20	N/A
Colorectal Cancer Resource & Action Network	20	N/A
Lymphoma Canada	20	N/A
Myeloma Canada	17	N/A
Canadian Breast Cancer Network	16	N/A
Canadian Skin Patient Alliance	13	N/A
Eczema Society of Canada	13	543,950
Save Your Skin Foundation	13	N/A
Arthritis Society Canada	12	N/A
Lung Cancer Canada	12	N/A
Lung Health Foundation / Ontario Lung Association	12	1,290,000
Diabetes Canada	11	710,000
Arthritis Consumer Experts	10	N/A
Bladder Cancer Canada	10	N/A
Breast Cancer Canada	10	N/A
Canadian Council of the Blind	10	N/A
Rethink Breast Cancer	10	N/A
Canadian Hemophilia Society	9	N/A
Eczéma Québec	9	N/A
Migraine Canada	9	N/A
Canadian Association of Psoriasis Patients	8	N/A
HeartLife Foundation	8	N/A
Kidney Cancer Canada	8	N/A
Kidney Foundation of Canada	8	N/A
Canadian Cancer Survivor Network	7	N/A
Canadian Spondyloarthritis Association	7	N/A
Fighting Blindness Canada	7	N/A
HAE Canada	7	N/A
HPV Global Action	7	N/A
Melanoma Canada	7	N/A
VTE-COS Patient Partners	7	N/A
Canadian Arthritis Patient Alliance	6	N/A
Oxalosis & Hyperoxaluria Foundation	6	N/A
Asthma Canada	5	N/A
British Columbia Lung Association and Lung Groups	5	N/A
Gastrointestinal Society	5	N/A
Migraine Quebec	5	N/A
Canadian Neuroendocrine Tumour Society	4	N/A
Lupus Ontario	4	N/A
Obesity Canada	4	N/A
Sickle Cell Awareness Group of Ontario	4	N/A
Women's Health Coalition of Alberta Society	4	N/A
Answering Thrombotic Thrombocytopenic Purpura Foundation	3	N/A
Cystic Fibrosis Canada	3	N/A
Leukemia & Lymphoma Society of Canada	3	N/A
Parkinson Association of Alberta	1	N/A
aHUS Canada	0	N/A
Aplastic Anemia & Myelodysplasia Association of Canada	0	N/A
Bardet Biedl Syndrome Foundation	0	N/A
Canadian Association of PNH Patients	0	N/A
Canadian Chronic Myelogenous Leukemia Network	0	N/A
Canadian FOP Network	0	N/A
Canadian Heart Patient Alliance	0	N/A
Canadian Psoriasis Network	0	N/A
Canadian VHL Alliance	0	N/A
Chronic Myelogenous Leukemia Society of Canada	0	N/A
CLL Canada	0	N/A
CreakyJoints Canada	0	N/A
Crohn's and Colitis Canada	0	N/A
Lupus Canada	0	N/A
Menopause Chicks	0	N/A
Mood Disorders Society of Canada	0	N/A
MS Canada	0	N/A
Muscular Dystrophy Canada	0	N/A
Obesity Matters	0	N/A
Parkinson Canada	0	N/A
Thalassemia Foundation of Canada	0	N/A
Thyroid Cancer Canada	0	N/A
TTR Amyloidosis Canada	0	N/A
Tumour Foundation of BC	0	N/A
Vasculitis Foundation Canada	0	N/A
Waldenstrom's Macroglobulinemia Foundation of Canada	0	N/A
Total (median, IQR)	451 (8, IQR 5, 12)	

**Table 4.** Patient Groups’ Website: Statement of Use of Payments from Pharmaceutical Companies.

Name of patient group	Use of payment
Answering Thrombotic Thrombocytopenic Purpura	Funding vital TTP research, providing educational materials and support programs
Bladder Cancer Canada	Patient education and awareness programs
Diabetes Canada	Support of 2023 Vascular Conference; Efforts in the areas of awareness, advocacy, public policy, education and camp
Gastrointestinal Society	Education
HeartLife Foundation	Unrestricted educational grants
Kidney Cancer Canada	Unrestricted grants
Kidney Foundation of Canada	Production of Living with Kidney Failure handbook
Lymphoma Canada	Educational webinars; Support of National Conference and Francophone Conference

### Payments Declared by Companies on Their Websites

The websites of five companies contained the value of payments to 34 patient groups that made submissions to CADTH—four companies disclosed payments made during 2023 and one during 2021. Payments ranged from $930 to the Canadian Association of Psoriasis Patients to almost $450,000 to Muscular Dystrophy Canada (median $80903, IQR 38838, 150906 (see Table 5). All the companies gave some information about the nature of the payments with GlaxoSmithKline providing the most detail.

**Table 5.** Company Payments to Patient Groups Based on Data Released by Pharmaceutical Companies.

Patient group	GlaxoSmithKline 2023	Merck 2021	Novartis 2023	Roche 2023	Sanofi 2023	Total revenue
	https://ca.gsk.com/media/7047/patient_organisation_funding_disclosure-2023-en.pdf	https://www.msd.com/wp-content/uploads/sites/9/2022/11/Canada-Grants-2021.pdf	https://www.novartis.com/sites/novartis_com/files/patient-organization-funding-report-2023.pdf	https://www.roche.com/about/sustainability/collaboration-with-patients/groups	https://www.sanofi.com/assets/dotcom/pages/docs/your-health/patient-support/Transparency-Report/PAGs-Transparency-Report-2023.pdf	
	Amount (CAN $)	Amount (CAN $)	Amount (CAN $)	Amount (CAN $)	Amount (CAN $)	Amount (CAN $)
Arthritis Consumer Experts			123,000			123,000
Arthritis Society			5,000		5,000	5,000
Asthma Society	110,000		10,000	1,525.29	105,000	226,525.29
Breast Cancer Canada			48,000			48,000
Canadian Association of Psoriasis Patients			930			930
Canadian Breast Cancer Network			61,782.5			61,782.5
Canadian Cancer Survivor Network	20,000	20,000	30,000		20,000	90,000
Canadian Council for the Blind			80,000			80,000
Canadian Chronic Myelogenous Leukemia Network			29,634.6			29,634.6
Canadian Hemophilia Society				388.61	90,000	90,388.61
Canadian Organization for Rare Disorders	15,000		50,000	2,675.3	65,000	132,675.3
Canadian Skin Patient Alliance			22,500		50,000	72,500
Canadian Spondylitis Association			20,000			20,000
Crohn's and Colitis Canada			145,000			145,000
Colorectal Cancer Canada	65,000	50,000				115,000
Colorectal Cancer Resource and Action Network	15,000	10,000	20,000	35,000		80,000
Diabetes Canada			25,000		96,000	121,000
Eczema Quebec					40,000	40,000
Eczema Society of Canada					100,000	100,000
Fighting Blindness Canada			155,000	195,093.23		350,093.23
HeartLife Foundation			56,170			56,170
Kidney Foundation of Canada	25,000					25,000
Leukemia and Lymphoma Society of Canada	10,000		10,000	15,000		35,000
Lung Cancer Canada		350			35,000	35,350
Lung Health Foundation/Ontario Lung Association	57,000	35,350	50,000		90,000	232,350
Lupus Canada	50,000					50,000
Lupus Ontario	10,000					10,000
Lymphoma Canada		20,000	30,000	31,805.06		81,805.06
Melanoma Canada		25,000	20,000		125,000	170,000
MS Canada			58,000		15,000	73000
Muscular Dystrophy Canada		15,000	383,500	1,009.94	50,000	449,509.94
Myeloma Canada	90,000				157,000	247,000
Rethink Breast Cancer		30,000	100,620	84,109.75		214,729.75
Save Your Skin Foundation		50,000		48,625	70,000	168,625

### Discordance Between Information in CADTH Submissions, on Patient Groups’ Websites and on Companies’ Websites

Twenty-seven patient groups had audited financial statements on their websites. Median annual total revenue per patient group was $1,721,218 (IQR 651785, 1118793). Based on data in patient groups’ declarations to CADTH, the estimated percent of total patient group revenue that came from companies ranged from 0.6 percent (Breast Cancer Canada) to 79.0 percent (Canadian Breast Cancer Network; median for all groups was 5.9%, IQR 1.1, 27.9). For the three patient groups where data about company payments were available on their websites, the estimated percent was: 2.1 percent (Diabetes Canada), 60.9 percent (Eczema Society of Canada), and 18.4 percent (Lung Health Foundation/Ontario Lung Association). Calculations based on data from the five companies that disclosed payments on their websites ranged from a low of 0.02 percent of the total revenue of Arthritis Canada up to a high of 20 percent for Rethink Breast Cancer (median 4.8%, IQR 2.3, 9.5) (see Table 6).

**Table 6.** Comparison Between CADTH Declarations, Information on Groups’ Websites and Data Released by Pharmaceutical Companies: Percent of Revenue from Company Payments.

Patient group name	Annual revenue (audited financial statement on groups’ websites) (CAN $)	Financial reporting year	Percent revenue from companies (from CADTH declarations)	Percent revenue from sponsor companies (from patient groups’ website)	Percent revenue from companies (from five companies’ websites)	URL for financial statement
Diabetes Canada	34,547,000	2023	6.1	2.1	0.4	https://www.diabetes.ca/getmedia/df11ff17-c704-41a8-ade2-4a801ea9b5fc/Diabetes-Canada-2023-FS-Final.pdf
Arthritis Society Canada	28,992,409	2023	1.3	N/A	0.02	https://arthritis.ca/getmedia/5162fc10-b653-42f2-b8e2-6348a5cec57e/2023-03-31-Arthritis-Society-18645-AUD-ASNPO.pdf
Breast Cancer Canada	17,193,789	2023	0.6	N/A	0.28	https://apps.cra-arc.gc.ca/ebci/hacc/srch/pub/dsplyRprtngPrd?q.srchNmFltr = breast + cancer + society&q.stts = 0007&selectedCharityBn = 137969861RR0001&dsrdPg = 1
Cystic Fibrosis Canada	13,792,000	2023	0.9	N/A	N/A	http://www.cfopn.org/uploads/1/9/3/3/19339065/financial_statement_2022_audited.pdf
Crohn's and Colitis Canada	13,586,259	2022	1.1	N/A	N/A	https://crohnsandcolitis.ca/Crohns_and_Colitis/documents/Crohn-s-and-Colitis-Audit-2022-Issued-FS.pdf
Parkinson Canada	13,149,557	2023	0.4	N/A	N/A	https://www.parkinson.ca/wp-content/uploads/Parkinson-Canada-Inc-FS-2023.pdf
Muscular Dystrophy Canada	11,189,793	2023	0.9	N/A	4.0	https://muscle.ca/wp-content/uploads/2023/10/Muscular_Dystrophy_Canada_12312023_Final_FSEnglish.pdf
Fighting Blindness Canada	7,665,486	2022	2.6	N/A	4.6	https://www.fightingblindness.ca/wp-content/uploads/2023/06/Audited-financial-statements-2022.pdf
Lung Health Foundation/Ontario Lung Association	6,993,675	2023	1.9	18.4	3.3	https://operationfresh.wpenginepowered.com/wp-content/uploads/2023/06/2023-03-31-Ont-Lung-Assn-14728-AUD-ASNPO.pdf
Myeloma Canada	4,219,519	2022	18.7	N/A	5.9	https://myeloma.ca/wp-content/uploads/2023/10/2022_fs_myeloma_canada_signed_by_bdo.pdf
Canadian Council of the Blind	3,099,238	2022	3.2	N/A	2.6	https://ccbnational.net/shaggy/wp-content/uploads/2024/02/Year-Ended-December-31-2022.pdf
Colorectal Cancer Canada	2,386,635	2023	39.0	N/A	4.8	https://www.colorectalcancercanada.com/wp-content/uploads/2024/01/Financial-Information-Colorectal-Cancer-Canada-20230630.pdf
Oxalosis & Hyperoxaluria Foundation	2,122,780	2020	2.4	N/A	N/A	https://ohf.org/wp-content/uploads/2022/02/2020-Final-Audited-Financials-OHF-12-31-2020-Final.pdf
Parkinson Association of Alberta	1,721,218	2023	0.4	N/A	N/A	https://parkinsonassociation.ca/wp-content/uploads/2023/04/Parkinsons-Association-of-Alberta-2023-Financial-Statements.pdf
Canadian Hemophilia Society	1,685,777	2023	21.5	N/A	5.4	https://www.hemophilia.ca/wp-content/uploads/2024/06/2023-CHS-financial-statements-FINAL-EN.pdf
Melanoma Canada	1,464,794	2023	6.8	N/A	11.6	https://melanomacanada.ca/wp-content/uploads/2024/03/2023-Financial-Statements-Melanoma-Canada.pdf
Lymphoma Canada	1,134,984	2023	32.6	N/A	7.2	https://www.lymphoma.ca/wp-content/uploads/2024/06/Lymphoma-Canada-FS-A23-FINAL54538.pdf
Mood Disorders Society of Canada	1,132,699	2023	23.8	N/A	N/A	https://mdsc.ca/wp-content/uploads/2023/12/MDSC-Audited-Mar-31-2023-Financial-statements.pdf
Rethink Breast Cancer	1,074,078	2023	27.9	N/A	20.0	https://rethinkbreastcancer.com/wp-content/uploads/2023/10/Rethink-Breast-Cancer-Canada-2023-03-31-FINAL-financial-statements.pdf
Eczema Society of Canada	893,628	2023	5.6	60.9	11.2	https://apps.cra-arc.gc.ca/ebci/hacc/srch/pub/t3010/v27/t3010Schdl6_dsplyovrvw
Gastrointestinal Society	651,785	2020	53.7	N/A	N/A	https://badgut.org/wp-content/uploads/GI-Society-Financial-Statements-2020-12-31-AUDITED.pdf
Lupus Canada	635,440	2022	9.4	N/A	7.9	https://www.lupuscanada.org/wp-content/uploads/2024/04/2022-Signed-Financial-Statements-March-1-2023-SIGNED.pdf
Lupus Ontario	505,835	2023	5.9	N/A	2.0	https://www.lupusontario.org/wp-content/uploads/2024/04/LO-Annual-Report-2023.pdf
Kidney Cancer Canada	471,109	2023	63.1	N/A	N/A	https://apps.cra-arc.gc.ca/ebci/hacc/srch/pub/dsplyRprtngPrd?q.srchNm = kidney + cancer + canada&q.stts = 0007&selectedCharityBn = 821670155RR0001&dsrdPg = 1
Canadian Breast Cancer Network	447,081	2023	79.0	N/A	11.1	https://www.cbcn.ca/web/default/files/public/Annuals%20and%20Financials/Audited%20Financial%20Statements%20-%20June%202023.pdf
Tumour Foundation of BC	241,976	2023	1.0	N/A	N/A	https://www.tumourfoundation.ca/annual-report/
Bardet Biedl Syndrome Foundation	156,492	2021	32.0	N/A	N/A	https://static1.squarespace.com/static/5e32341cff63c7446f3fdf1d/t/6399f43fa9e1183a126e25cf/1671033919254/Bardet + Biedl + Syndrome + Foundation + 990-EZ + FY + 2021.pdf
Median (IQR)	1,721,218 (651785, 1118793)		5.9 (1.1, 27.9)		4.8 (2.3, 9.5)	

The percent of revenue from companies for some patient groups appeared similar independent of whether the information came from CADTH declarations, patient groups’ websites or companies’ websites. For example, based on CADTH declarations, the Canadian Council of the Blind received 3.2 percent of its revenue from companies and 2.6 percent based on data in companies’ websites. For other patient groups, the percent was widely divergent; for example, based on CADTH declarations, the Eczema Society of Canada received 5.6 percent of its revenue from company sources, 60.9 percent based on information on its website and 11.2 percent based on companies’ websites.

Seventeen groups declared COI in their submissions to CADTH, but they did not mention any company sponsors on their websites, whereas four groups listed company sponsors on their websites but did not declare any COI to CADTH. For example, the Canadian Psoriasis Network declared 13 COI to CADTH but did not provide the names of any company sponsors on its website, while Arthritis Consumer Experts listed 10 company sponsors on its website but declared none to CADTH in its four submissions. Even when patient groups gave information about company relationships on their websites and CADTH submissions, there were still major discrepancies in the numbers. For example, Colorectal Cancer Canada declared two COI to CADTH but named 17 company sponsors on its website. On the other hand, the Arthritis Society Canada declared 17 COI to CADTH but only 12 sponsors were listed on its website. Eight groups listed no COI on either their websites or in their CADTH submissions.

## Discussion

The most important conclusion from this study is that obtaining transparent information about Canadian patient group funding is not possible given the multiple limitations in data on the number of COI (pharmaceutical company sponsors) that Canadian patient groups have and the amount of money that they receive from them. Poor reporting of patient groups’ COI and revenue from pharmaceutical companies is not confined to Canada. Only 25 percent of American health advocacy organizations that received Eli Lilly grants acknowledged Lilly's contributions on their websites, and only 10 percent acknowledged Lilly as a grant event sponsor. No organization disclosed the exact amount of a Lilly grant.^20^ In Australia, only 52.3 percent of patient groups acknowledged that they had received company donations, 13.2 percent disclosed the amount, and 4.4 percent communicated the proportion of their income that came from industry.^21^ This study confirms that the question of where patient groups get their income from and how much they receive is not confined to individual countries but has international importance.

Rickard and colleagues^4^ have enumerated the three general dimensions associated with transparency: availability of information, accuracy of information, and clarity of information. Together the three indicators form the fundamental bases for informed, evidence-based decision making. Transparency about payments that patient groups in Canada receive is lacking in all three dimensions. Information from CADTH declarations and on patients’ and companies’ websites is incomplete, inconsistent, unreliable, and unclear. One of the reasons for the incompleteness and inconsistency is that CADTH only requires declarations when patient groups have a direct or indirect interest relationship with companies that make the drug under review. CADTH leaves it to the groups themselves to reach a decision on this question and groups may have different decision-making criteria. CADTH's practice of deferring to patient groups to judge what is and is not relevant mirrors the policy of many medical journals, which is a major contributory factor behind the inconsistent and incomplete COI declarations frequently seen in journals.^22^

However, even allowing for ambiguity about what a direct or indirect interest may be, there are still major discrepancies in our knowledge about patient groups’ COI and funding that are difficult to explain, such as Colorectal Cancer Resource & Action Network declaring two COI to CADTH but naming 20 company sponsors on its website. Trying to compare the percent of total revenue coming from companies based on different sources of information is unreliable for a variety of reasons: CADTH disclosures are incomplete, CADTH groups the value of payments into broad categories, patient groups’ websites provide little information about funding, and only a small minority of companies report payments. One further explanation for the different estimates may be that information on the different sites covers different time periods, but this reason just emphasizes the problem in obtaining accurate and complete data and accentuates a lack of trust in the reporting by both patient groups and pharmaceutical companies.

The lack of transparency about patient groups’ COI and the amount of money that they received as a result of their relationships with pharmaceutical companies means that when groups take positions on controversial issues, it is difficult to know if they are speaking on behalf of their membership or if their statements might be influenced by their financial relationships with the companies that support them. This lack of certainty threatens the credibility of patient groups.^23^ Examples of the ambiguity surrounding their allegiance have occurred in the United States, in Europe, and in Canada. In the United States, the Centers for Medicare and Medicaid Services proposed a project aimed at lowering spending on the most expensive treatments offered under Medicare Part B, the federal insurance plan that covers outpatient drug costs. One hundred and forty-seven patients’ groups signed letters opposing the project, 110 of which received funding from the pharmaceutical industry.^24^ The Epilepsy Foundation, which has representatives from four companies on its board that market medications for epilepsy and that provide funding for the foundation, campaigned for bills introduced in U.S. state legislatures that would make it harder for pharmacists to substitute generic drugs for brand name epilepsy medications.^25^ A survey of patient and consumer groups in Europe found an association between receiving industry sponsorship and support for an expanded role for the pharmaceutical industry as an information provider to the public about the products that companies make.^26^ In Canada, the Canadian Organization for Rare Disorders (CORD) has publicly criticized the legislation that potentially creates the first steps to a universal, first-dollar coverage pharmacare plan.^27^ Further, in 2018 its CEO told the *Globe and Mail* that since she does not know what the right price for a drug should be, she does not believe that she should be advocating for drug companies to either raise or lower their prices.^28^ CORD has also been criticized by CADTH for failing to disclose in a submission that it had a COI within the previous two years with a company that was applying for reimbursement for a drug for a rare disorder.^29^

Pharmaceutical companies disproportionately fund patient groups operating in therapeutic areas relevant to the drugs that they are marketing.^30^ in the hope that patient groups will amplify their message about the benefits and safety of their drugs.^31^ Without transparency about the source of funding, it is not possible to identify a COI situation and distinguish between a group's genuine belief in the value of a product or whether it is uncritically endorsing the company's position. Equally as important, this bias in making funding available to only certain groups can have the effect of drowning out voices that express skepticism about a drug and its price. Finally, as Bero and Parker note, “industry sponsorship could give companies direct access to patients through their attendance at industry-sponsored events or participation in industry-sponsored support groups. This access could be used to gather information for marketing or for new types of promotion, such as through social media”.^32^^ p.75^

Questions about whether any level of industry funding is acceptable and if it is, whether there should be a maximum overall amount from industry in general and from single companies in particular are still being debated. In Australia, Parker and colleagues convened a nationwide meeting of health consumer organizations (HCOs) to discuss the risks and benefits of partnering with the pharmaceutical industry and “to work towards a set of principles and suggestions for best practice that HCOs might draw on when considering whether, or how, to interact with pharmaceutical industry funders”.,^33^^p. 393^

While this conversation needs to continue on a national and international level, there are concrete measures that can be taken while that conversation is ongoing. A first necessary, but not sufficient, response to the lack of transparency is disclosure of information. Therefore, it should be incumbent on patient groups to maintain an up-to-date and accurate list of the names of companies that they receive donations from, the year that those donations were made, and the amount of the donation. However, voluntary disclosure of COI and funding by patient groups is usually scarce as the situation in Australia,^21^ Italy,^11^ the United Kingdom,^10^ and the United States^20^ has amply demonstrated. Legislation rather than voluntarism is what is needed. Before the Ontario election in 2019, the government was finalizing regulations for Bill 160, which required all drug and device manufacturers that provided a “transfer of value” to individual health care practitioners and health care organizations, including patient groups, to report those transfers to a public registry.^34^ The legislative process stopped when the government changed post-election. The federal government should pick up the mandate on this issue and pass similar legislation to make reporting mandatory on a national basis.

### Limitations

There was no way to verify that COIs declared to CADTH were contemporaneous with the name of sponsors listed on groups’ websites and the names of patient groups that companies disclosed on their websites. An attempt was made to ensure that the information in all three places was consistent by using the most recently available information on all three sites. Some patient groups’ websites had password protected member sites and additional information about company sponsors may have been provided to people with site permissions. The results of this study only apply to patient groups making submissions to CADTH from the start of 2022 to September 2023 and may not be relevant for other patient groups. Since the data were gathered by a single individual, inadvertent errors may have been introduced.

## Conclusion

At present, information about Canadian patient groups’ COI and funding is extremely limited as is the case in other countries where these relationships have been studied. To retain confidence that these groups are acting in the best interests of their membership, greater transparency about the payments that they receive from companies is a necessary first step. Voluntary disclosure is not sufficient, and legislation is necessary.

## Supplemental Material

sj-xlsx-1-joh-10.1177_27551938251325801 - Supplemental material for Information About Canadian Patient Groups’ Conflicts of Interest and Industry Funding—Incomplete, Inconsistent, and Unreliable: A Cross-Sectional Study Supplemental material, sj-xlsx-1-joh-10.1177_27551938251325801 for Information About Canadian Patient Groups’ Conflicts of Interest and Industry Funding—Incomplete, Inconsistent, and Unreliable: A Cross-Sectional Study by Joel Lexchin in International Journal of Social Determinants of Health and Health Services

sj-docx-2-joh-10.1177_27551938251325801 - Supplemental material for Information About Canadian Patient Groups’ Conflicts of Interest and Industry Funding—Incomplete, Inconsistent, and Unreliable: A Cross-Sectional Study Supplemental material, sj-docx-2-joh-10.1177_27551938251325801 for Information About Canadian Patient Groups’ Conflicts of Interest and Industry Funding—Incomplete, Inconsistent, and Unreliable: A Cross-Sectional Study by Joel Lexchin in International Journal of Social Determinants of Health and Health Services
