# Supplementary material for: Information About Canadian Patient Groups’ Conflicts of Interest and Industry Funding—Incomplete, Inconsistent, and Unreliable: A Cross-Sectional Study
Source: Int J Soc Determinants Health Health Serv. 2025 Mar 17;55(3):352–67. doi: 10.1177/27551938251325801 (PMC12171057; doi:10.1177/27551938251325801)
Supplement: sj-docx-2-joh-10.1177_27551938251325801 - Supplemental material for Information About Canadian Patient Groups’ Conflicts of Interest and Industry Funding—Incomplete, Inconsistent, and Unreliable: A Cross-Sectional Study [file sj-docx-2-joh-10.1177_27551938251325801.docx]

Supplementary Table 2: Comparison of conflict-of-interest declarations by same patient group in different submissions to CADTH

| **Patient group** | **Pharmaceutical companies** | | | | | | | |
| --- | --- | --- | --- | --- | --- | --- | --- | --- |
| **Aplastic Anemia & Myelodysplasia Association of Canada** | Alexion, Bristol Myers Squibb, Novartis, Sobi TAIHO Takeda | Alexion, Bristol Myers Squibb, Novartis, Sobi, Taiho, Takeda |  |  |  |  |  |  |
| **Arthritis Consumer Experts** | No COI | No COI | No COI | Abbvie |  |  |  |  |
| **Arthritis Society Canada** | Abbvie, Alcon, Amgen, Boehringer Ingelheim, Bristol Myers Squibb, Celgene, Eli Lilly, Eupraxia Pharmaceuticals, Gilead, Johnson+Johnson, Janssen, Merck, Novartis, Pfizer, Sanofi, UCB | Abbvie, Alcon, Amgen, Boehringer Ingelheim, Bristol Myers Squibb, Celgene, Eli Lilly, Eupraxia Pharmaceuticals, Gilead, Johnson+Johnson, Janssen, Merck, Novartis, Pfizer, Sanofi, UCB | Abbvie, Amgen, Boehringer Ingelheim, Bristol Myers Squibb, Eli Lilly, Johnson+Johnson, Janssen, Merck, Novartis, Pfizer, Valeo |  |  |  |  |  |
| **Asthma Canada** | AstraZeneca, GlaxoSmithKline, Novartis, Pfizer, Sanofi, Teva Valeo | AstraZeneca, GlaxoSmithKline, Novartis, Pfizer, Sanofi | AstraZeneca, GlaxoSmithKline, Novartis, Pfizer, Sanofi |  |  |  |  |  |
| **Canadian Arthritis Patient Alliance** | Abbvie, Janssen, UCB | Abbvie, Janssen, UCB | Abbvie, Janssen, UCB | Abbvie, Janssen, UCB |  |  |  |  |
| **Canadian Association of Psoriasis Patients** | Abbvie, Amgen, Bausch, Boehringer Ingelheim, Bristol Myers Squibb, Janssen, LEO, Novartis, Pfizer, Sun, UCB | Abbvie, Amgen, Bausch, Janssen, Novartis, Pfizer, Sun, UCB | Abbvie, Amgen, Arcutis, Bausch, Boehringer Ingelheim, Janssen, LEO, Novartis, Pfizer, Sun, UCB |  |  |  |  |  |
| **Canadian Breast Cancer Network** | Eli Lilly | Novartis | Roche | AstraZeneca | Merck | Merck | AstraZeneca, Eli Lilly, Gilead, Janssen, Merck, Novartis, Pfizer, Roche |  |
| **Canadian Cancer Survivor Network** | Amgen, Bayer, Janssen | AstraZeneca | Novartis | Janssen | AstraZeneca | Merck | Merck |  |
| **Canadian Council of the Blind** | Bayer, Novartis | Bayer, Novartis | Bayer, Novartis | Bayer, Novartis |  |  |  |  |
| **Canadian Liver Foundation** | AstraZeneca, Bristol Myers Squibb, Eisai, Ipsen, Roche | Roche | No COI |  |  |  |  |  |
| **Canadian Organization for Rare Disorders** | Ultragenyx | Alnylam Pharmaceuticals | Ipsen | AstraZeneca/Alexion | Novo Nordisk |  |  |  |
| **Canadian Psoriasis Network** | Abbvie, Amgen, Bausch, Boehringer Ingelheim, Bristol Myers Squibb, Janssen, LEO, Novartis, Pfizer, Sun, UCB | Abbvie, Amgen, Bausch, Boehringer Ingelheim, Bristol Myers Squibb, Janssen, LEO, Novartis, Pfizer, Sun, UCB | Abbvie, Amgen, Arcutis, Bausch, Boehringer Ingelheim, Bristol Myers Squibb, Janssen, LEO, Novartis, Pfizer, Pierre Fabry, Sun, UCB |  |  |  |  |  |
| **Canadian Skin Patient Alliance** | Abbvie, Janssen, Merck, Pfizer, Sanofi, UCB | Abbvie, Janssen, Merck, Novartis, Pfizer, Sanofi, UCB | Abbvie, LEO, Pfizer, Sanofi | Abbvie, Bausch, Boehringer Ingelheim, Bristol Myers Squibb, Janssen, LEO, Novartis, Pfizer |  |  |  |  |
| **CLL Canada** | AstraZeneca, BeiGene, Janssen | AstraZeneca, BeiGene, Janssen |  |  |  |  |  |  |
| **Colorectal Cancer Canada** | Abbvie, Amgen, AstraZeneca, Bayer, Boehringer Ingelheim, Bristol Myers Squibb, Elevation Oncology, Eli Lilly GlaxoSmithKline, Janssen, Merck, Novartis, Pendopharm, Pfizer, Roche, Taiho | Abbvie, Amgen, AstraZeneca, Bayer, Boehringer Ingelheim, Bristol Myers Squibb, GlaxoSmithKline, Incyte, Janssen, Merck, Novartis, Pendopharm, Pfizer, Roche, Taiho |  |  |  |  |  |  |
| **Colorectal Cancer Resource & Action Network** | No COI | Merck | Taiho |  |  |  |  |  |
| **CreakyJoints Canada** | Abbvie | Abbvie | Abbvie |  |  |  |  |  |
| **Crohn’s and Colitis Canada** | Eli Lilly | Bristol Myers Squibb | Abbvie | Abbvie | Abbvie |  |  |  |
| **Diabetes Canada** | AstraZeneca, Bayer, Janssen, Novo Nordisk, Sanofi | AstraZeneca, Bayer, Janssen, Novo Nordisk, Sanofi | AstraZeneca, Bayer, Janssen, Novo Nordisk, Sanofi | AstraZeneca, Bayer, Eli Lilly, Janssen, Novo Nordisk, Paladin, Sanofi, Takeda | Abbott, Ascensia, AstraZeneca, Boehringer Ingelheim, Dexcom, Eli Lilly, Insulet, Janssen, Lifescan Canada, Medtronic, Novo Nordisk, Sanofi, Takeda |  |  |  |
| **Fighting Blindness Canada** | Abbvie-Allergan, Bayer, Novartis, Roche | Abbvie-Allergan, Bayer, Novartis, Roche | Abbvie-Allergan, Bayer, Novartis, Roche | Bayer, Novartis, Roche |  |  |  |  |
| **Gastrointestinal Society** | AstraZeneca | Eli Lilly | Bristol Myers Squibb | Abbvie | Novo Nordisk | Abbvie | Abbvie |  |
| **HeartLife Foundation** | Bayer, Boehringer Ingelheim, Bristol Myers Squibb, Novartis, Servier | AstraZeneca, Boehringer Ingelheim | AstraZeneca, Bayer, Boehringer Ingelheim, Bristol Myers Squibb, Novartis, Servier | AstraZeneca, Bayer, Boehringer Ingelheim, Bristol Myers Squibb, Novartis, Servier |  |  |  |  |
| **Kidney Cancer Canada** | Bristol Myers Squibb, Eisai, Ipsen, Merck, Pfizer | Bristol Myers Squibb, Eisai, Ipsen, Merck, Novartis, Pfizer |  |  |  |  |  |  |
| **Kidney Foundation of Canada** | Alexion, Amgen, AstraZeneca, Bayer, GlaxoSmithKline, Horizon, Janssen, Otsuka, Paladin, Takeda | No COI | Alexion, Amgen, AstraZeneca, Bayer, Horizon, Janssen, Otsuka, Paladin, Takeda | Alexion, Amgen, ASTELLAS, AstraZeneca, Horizon, Janssen, Otsuka, Paladin, Takeda | Alexion, Amgen, AstraZeneca, Bayer, GlaxoSmithKline, Horizon, Janssen, Otsuka, Paladin, Takeda |  |  |  |
| **Leukemia & Lymphoma Society of Canada** | Novartis | Sanofi | Gilead | Servier | Jazz | Takeda | No COI | No COI |
| **Lung Cancer Canada** | Janssen | Roche | Roche | Roche | Jazz | Bristol Myers Squibb | Roche |  |
| **Lung Health Foundation / Ontario Lung Association** | Janssen | Roche | AstraZeneca | Jazz |  |  |  |  |
| **Lupus Canada** | AstraZeneca | AstraZeneca, GlaxoSmithKline |  |  |  |  |  |  |
| **Lymphoma Canada** | Bristol Myers Squibb, Gilead, Novartis | Bristol Myers Squibb, Gilead, Novartis | Bristol Myers Squibb, Gilead, Incyte, Novartis, Roche | AstraZeneca, BeiGene, Janssen | Bristol Myers Squibb, Gilead, Incyte, Novartis, Roche | Bristol Myers Squibb, Gilead, Novartis | AstraZeneca, BeiGene, Janssen |  |
| **Melanoma Canada** | Bristol Myers Squibb | Merck |  |  |  |  |  |  |
| **Migraine Canada** | Abbvie, Eli Lilly, Lundbeck, Miravo, Novartis, Teva | Abbvie, Eli Lilly, Lundbeck, Miravo, Novartis, Teva | No COI |  |  |  |  |  |
| **Migraine Quebec** | Allergan-Abbvie, Aralez/Novo/Miravo, Eli Lilly, Lundbeck, Novartis, Upjohn, Viatris | Allergan-Abbvie, Aralez/Novo/Miravo, Eli Lilly, Lundbeck, Novartis, Upjohn, Viatris |  |  |  |  |  |  |
| **Mood Disorders Society of Canada** | Abbvie, Eisai, Janssen, Lundbeck, Pfizer | Abbvie, Eisai, Janssen, Lundbeck, Pfizer, Viatris |  |  |  |  |  |  |
| **Muscular Dystrophy Canada** | Argenx | Alexion |  |  |  |  |  |  |
| **Myeloma Canada** | Abbvie, Amgen, Apotex, AstraZeneca, Binding Site, Bristol Myers Squibb, Cellpoint, Forus Therapeutics, GlaxoSmithKline, Janssen, JAMP, Karyopharm, Lonza, Merck, Novartis, Pfizer, Rapid Novor, Roche, Sanofi, Sebia, Takeda, Telo Genomics | Amgen, Bristol Myers Squibb, Celgene, GlaxoSmithKline, Janssen, Karyopharm, LEO, Merck, Novartis, Pfizer, Rapid Novor, Sanofi, Takeda |  |  |  |  |  |  |
| **Rethink Breast Cancer** | Eli Lilly | AstraZeneca | Merck | Merck | Gilead | AstraZeneca | AstraZeneca, Daiichi Sanyko |  |
| **Save Your Skin Foundation** | Bristol Myers Squibb | Merck | No COI |  |  |  |  |  |
| **VTE-COS Patient Partners** | AstraZeneca | Abbvie, GlaxoSmithKline, Janssen |  |  |  |  |  |  |
